# Thermal modeling of lesion growth with radiofrequency ablation devices

**DOI:** 10.1186/1475-925X-3-27

**Published:** 2004-08-06

**Authors:** Isaac A Chang, Uyen D Nguyen

**Affiliations:** 1 Office of Science and Engineering Laboratories, Center for Devices and Radiological Health, U.S. Food and Drug Administration, Rockville, Maryland, USA; 2 Department of Biomedical Engineering, Catholic University of America, Washington DC, USA

## Abstract

**Background:**

Temperature is a frequently used parameter to describe the predicted size of lesions computed by computational models. In many cases, however, temperature correlates poorly with lesion size. Although many studies have been conducted to characterize the relationship between time-temperature exposure of tissue heating to cell damage, to date these relationships have not been employed in a finite element model.

**Methods:**

We present an axisymmetric two-dimensional finite element model that calculates cell damage in tissues and compare lesion sizes using common tissue damage and iso-temperature contour definitions. The model accounts for both temperature-dependent changes in the electrical conductivity of tissue as well as tissue damage-dependent changes in local tissue perfusion. The data is validated using excised porcine liver tissues.

**Results:**

The data demonstrate the size of thermal lesions is grossly overestimated when calculated using traditional temperature isocontours of 42°C and 47°C. The computational model results predicted lesion dimensions that were within 5% of the experimental measurements.

**Conclusion:**

When modeling radiofrequency ablation problems, temperature isotherms may not be representative of actual tissue damage patterns.

## Introduction

The mitigation of primary and metastatic tumors by radiofrequency ablation is a developing research area. The goal of ablation is to necrose treatment volumes by raising the temperature of targeted tissues. Ablation probes are inserted percutaneously, laparoscopically, or during surgery into cancerous tumors. Once positioned, high frequency alternating current (450–550 kHz) is delivered through an uninsulated electrode into the surrounding tissues to a dispersive ground pad that is applied to the patient. The electromagnetic energy is converted to heat by resistive heating.

While the usage of radiofrequency ablation devices is well established, efforts to optimize treatment strategies are ongoing. An important consideration in optimizing ablation is determining what treatment volumes are necessary and acceptable. In liver ablation, for example, treatment volumes generally extend a centimeter beyond the dimensions of a tumor [1-3]. Since the liver possesses regenerative characteristics, it is more critical to insure that necrosis is achieved in 100% of the cancerous cell volume than to minimize damage to healthy tissues. In contrast, a centimeter margin in cardiac ablation is generally unacceptable since many vital substructures are in close proximity.

The growth of ablation lesions remains a central issue in the development of radiofrequency ablation devices. Knowing the expected shape of lesions is essential for treatment planning and procedure optimization. To date, many approaches have been attempted to characterize lesion size. The results have varied widely. Ablation lesions generated *in vitro *and *in vivo *in animal models show wide variations, since many of the key parameters (i.e. tissue perfusion) cannot be controlled [4,5]. In addition, the boundaries of lesions in animal models are often "fuzzy" and are subject to interpretation. Computational modeling is a valuable tool in the optimization process, since it allows the systematic examination of the various parameters affecting the outcome of ablation. However, most computational models fail to capture essential physiologic phenomena.

Many computational studies have been reported in the literature to predict the growth of lesion size during ablation [6-19]. However, the majority of these models do not directly calculate lesion size. Surrogate endpoints, such as temperature [20-22] and thermal dosing [23] are calculated and are interpreted as being equivalent to lesion size. In many cases, these surrogate endpoints do not correlate well with clinical outcome and vary considerably. The microwave hyperthermia literature, for example, cites 42 degrees Celsius as the point at which thermal damage occurs to tissues [24,25]. In the cardiac ablation literature, 47 degrees Celsius is generally accepted as the onset of tissue damage [23,24,26,27]. Neither of these values can be derived directly from gross histological measurements of lesion size, since the tissue pathology does not provide a record of temperature. Many computational studies justify these surrogate endpoints by showing a high correlation between temperature isotherms and lesion size. However, temperature isotherms and lesion size have never actually been shown to be equivalent.

Several investigators have demonstrated that tissue damage is a function of both temperature and time [28-30]. As tissue temperature is increased, the amount of time necessary to achieve a threshold of damage decreases. Tissue damage can be characterized using the Arrhenius equation which relates temperature and exposure time using a first order kinetics relationship. Data from experimental studies, where tissues are exposed to uniform temperatures for controlled time intervals, are fit to the Arrhenius equation to determine the frequency factor **A**(s^-1^), and the activation energy Δ**E **(J mol^-1^). Arrhenius parameters have been determined in skin [31-35], artery [36,37], blood [38-40], pancreas [41,42], heart [43], cornea [44-46], muscle [47], prostate [48], ovary [49], kidney [50-52], and liver [30,52,53]. For a specified exposure temperature and time, the fit parameters **A **and Δ**E **determine the amount of cell damage incurred for a specific tissue type. In combination with computational modeling techniques, it is then possible to calculate the distribution of cell damage surrounding ablation probes.

In this study, we compare the temperature distribution and tissue necrosis patterns for a hepatic ablation probe at body temperatures. At each time step, the specific absorption rate (SAR), temperature, and the tissue damage are calculated. The level of tissue perfusion is varied for the models to determine the maximum variation in lesion size resulting from a typical ablation. These data are validated experimentally using an ablation probe in liver tissue.

## Methods

Radiofrequency ablation probes operate between 460–550 kHz. At these frequencies, the wavelength of the electromagnetic energy is several orders of magnitude larger than the size of the ablation electrodes. Thus, the primary mode of energy transfer is through electrical conduction and can be modeled as a coupled quasistatic electrical conduction and heat conduction problem. The electric field is solved by using Laplace's equation,

∇·[*σ*(*T*)∇*V*] = 0     (Eq.1)

where ∇ is the gradient operator, σ (T) is the temperature-dependent conductivity (Siemens/meter), and V is the electric potential (Volts). Temperature is solved by using a modified Pennes bioheat equation [54],



where ρ is the density, 1060 kg/m^3 ^[55]; **C **is the heat capacity of tissues, 3600 J/kg-K [55]; **k **is the heat conduction coefficient, 0.502 W/K-m [55]; ρ_b _is the density of blood, 1000 kg/m^3 ^[9]; C_b _is the heat capacity of blood, 4180 J/kg-K [9];α is a tissue state coefficient; ω is the blood perfusion coefficient, 6.4 × 10^-3 ^sec^-1 ^[9];T_amb _is the ambient body temperature, 37°C; and Q_m _is the metabolic heat source term. For all cases, we assumed that the metabolic heat source was insignificant. The tissue state coefficient (α) ranges from 0–1 depending on the local level of tissue damage

At each time step, the cumulative damage integral is computed using the well established Arrhenius equation



where Ω(t) is the degree of tissue injury, *c(t) *is the concentration of living cells, *R *is the universal gas constant, *A *is a "frequency" factor for the kinetic expression (s^-1^), and Δ*E *is the activation energy for the irreversible damage reaction (J-mol^-1^) [50]. The kinetic parameters account for morphologic changes in tissue relating to the thermal degradation of proteins [56]. The parameters *A *and Δ*E *are dependent on the type of tissue and have been characterized for liver tissues by Jacques et. al. (A = 7.39 × 10^39 ^s^-1 ^and ΔE = 2.577 × 10^5 ^J-mol^-1^) [52]. In the context of finite element modeling of tissue damage, a damage integral of Ω = 1, corresponds to a 63% percent probability of cell death at a specific point. A damage integral of Ω = 4.6, corresponds to 99% percent probability of cell death at a point in the model. The significance of Ω = 1 has been reported as the point at which tissue coagulation first occurs [36]. Once tissue coagulation occurs, tissue perfusion ceases. This corresponds to a tissue state coefficient of α = 0. Intermediate levels of the tissue state coefficient are calculated as α = 1/exp(Ω).

**Figure 1:**
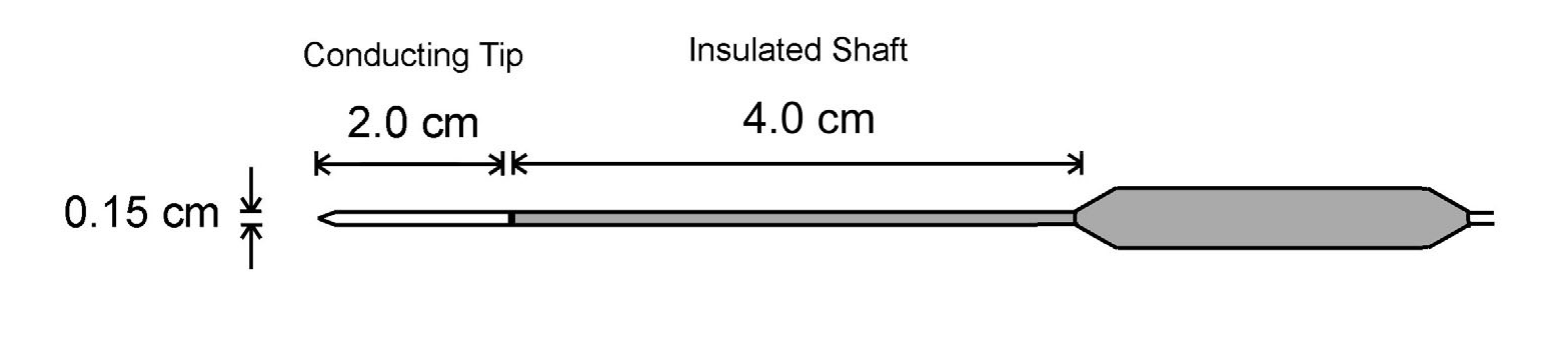
Ablation probe geometry diagram of a single needle ablation electrode that is used for hepatic tumor ablation. Therapeutic treatment is achieved by applying a source voltage to the conducting tip. A conducting pad applied to the patient skin serves as an electrical ground return.

**Figure 2:**
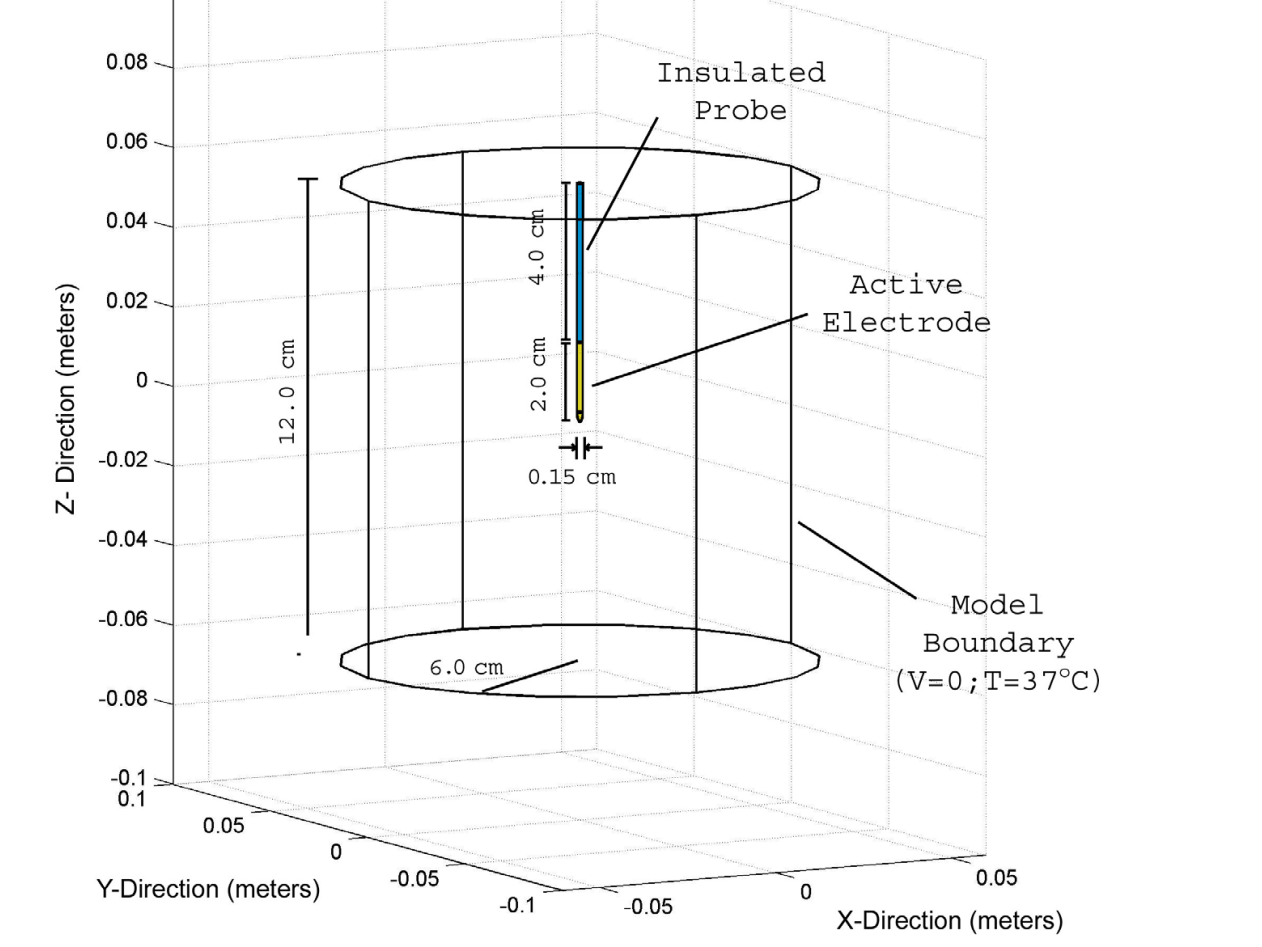
Model geometry three dimension representation of the axisymmetric two-dimensional finite element model. All external surfaces of the cylindrical model serve as the electrical ground and are at body temperatures (37°C). The entire ablation probe is assumed to be thermally insulating.

Figure 1 shows a diagram of a typical needle ablation electrode used in clinical practice for hepatic tumor ablation. The probe is 6.0 cm long with a diameter of 0.15 cm. The distal 2.0 cm of the probe is uninsulated and the proximal 4.0 cm of the probe is covered with a thin electrically insulating material. Figure 2 shows a three-dimensional representation of the axisymmetric two-dimensional geometry of the model. The active portion of the probe is situated in the center of a cylindrical model that is 6.0 cm in radius and 12.0 cm in height. Electrical and thermal properties of liver are used in the model to simulate a fully-embedded insertion of the needle electrode. The electrical properties of tissue are assumed to be temperature dependent and solved according to Chang [57], where the electrical conductivity appears as

σ(T,*N*) = σ(25,*N*) {1.000-1.962 × 10^-2^Δ + 8.08 × 10^-5^Δ^2^

- *N*Δ [3.020 × 10^-5 ^+ 3.922 × 10^-5^Δ + *N*(1.721 × 10^-5^Δ

- 6.584 × 10^-6^Δ)]}     (Eq.4)

where

σ (25,*N*) = *N *[10.394-2.3776 *N *+ 0.68258 *N*^2 ^- 9.13538 *N*^3 ^+ 1.0086 × 10^-2 ^*N*^4^]

;N is the normality of an electrically equivalent sodium chloride solution, *N *= 0.0111; and Ä = 25-T, which produces an equivalent electrical conductivity of liver tissues at 37°C (approximately 0.134 S/m). The thermal properties of liver used in the model were acquired from Tungjitkusolmun *et al.*[9] and Duck [55].

A source voltage (V_o_) is applied to the conducting tip of the ablation probe. The outer surface of the model serves as an electrical ground return (V = 0). An electrically insulating boundary condition is applied to the non-conducting portions of the probe such that **n**·(σ∇ V) = 0; where **n **is the unit vector normal to the surface, σ is the electrical conductivity, and V is the voltage at the insulating surface. A thermal boundary condition of T = T_amb _is applied to the outer surfaces of the model to simulate ambient temperature. Since the thermal mass of the probe is small compared to the surrounding tissue, we assumed that heat conduction into the probe itself was minimal. Thus, all other surfaces of the ablation probe are considered to have a thermally insulating boundary condition such that **n**·(k ∇ T) = 0.

**Figure 3:**
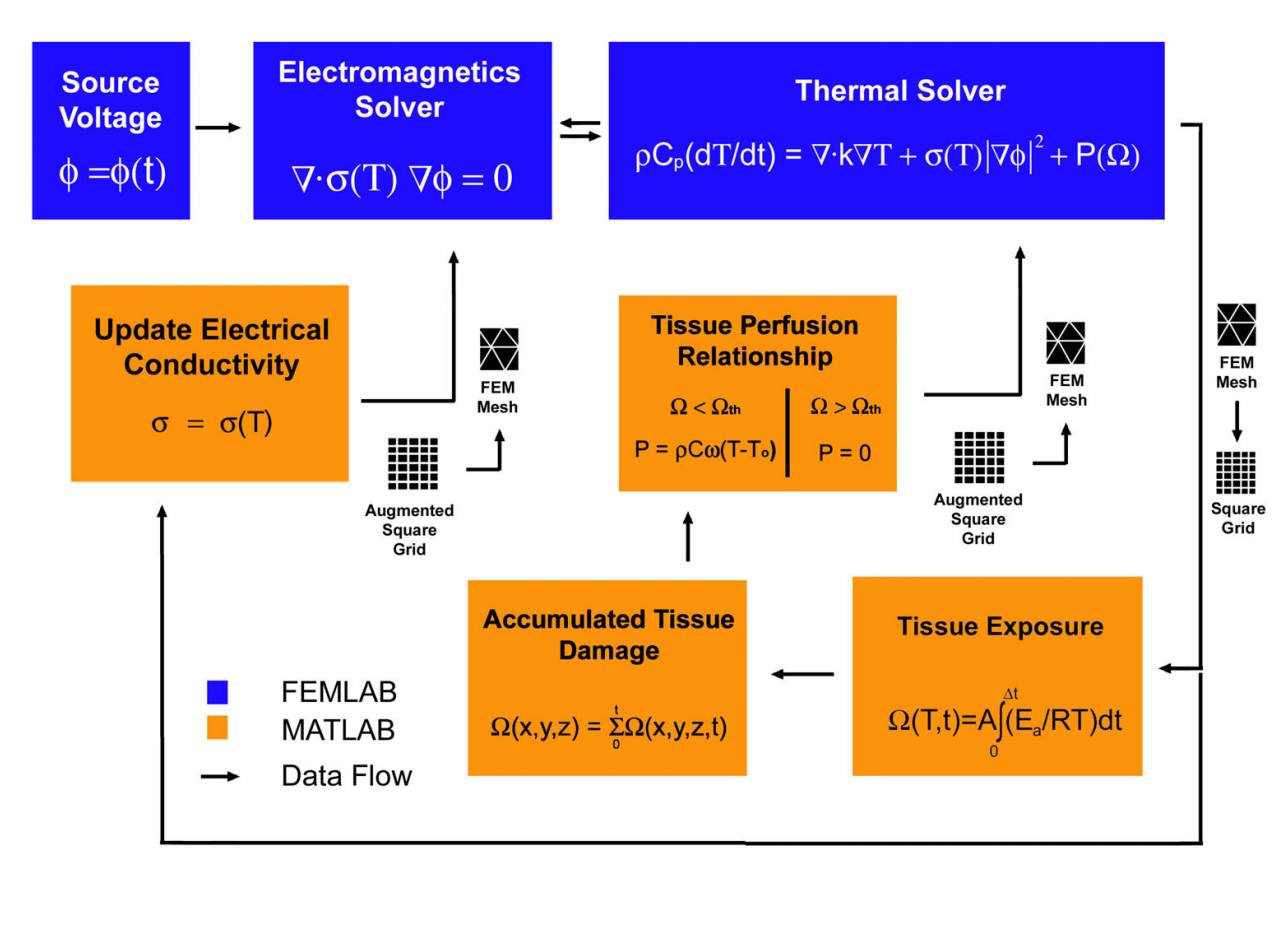
Computational technique diagram of data flow used in a hybrid finite element model implemented in Femlab/Matlab to calculate temperature and tissue damage. The electric field and temperature are solved simultaneously in Femlab (blue blocks). The data structure is changed from finite element meshing to rectilinear gridding so that the resulting temperature can be used to calculate tissue exposure and electrical conductivity change in Matlab (orange blocks). A tissue damage level of 63% corresponds to the onset of tissue necrosis and is associated with a cessation in local blood flow. The Matlab results are then imported into Femlab as inputs for calculation at the next time step.

A hybrid finite element model was developed using Femlab (Comsol, Burlington MA, USA) and Matlab (Mathworks, Natick MA, USA) to calculate temperature and tissue damage. While conventional finite element models effectively solve field solutions using a nonuniform geometrical mesh, tissue exposure calculations are integrated at each point in the model over the course of ablation and are more easily calculated using uniform rectilinear grids. As shown in Figure 3, Femlab is used to solve the coupled electromagnetic and heat conduction equations simultaneously at each timestep. This is done to insure that the temperature-dependent electrical conductivity is updated with each iterative calculation of temperature for a given timestep. The converged temperature is mapped from the finite element mesh into a rectilinear grid, which is passed into the Matlab environment. The amount of tissue damage occurring at each timestep is calculated using the Arrhenius equation and tracked at each point in the model. Once the level of damage exceeds 63% cell damage, it is assumed that tissue coagulation has occurred, causing a cessation in tissue perfusion. The 63% cell damage point is historically used because it corresponds to the earliest onset of visible tissue coagulation. A rectilinear grid containing the perfusion characteristics at each point in the model is mapped back into the finite element mesh and used in subsequent Femlab calculations. The rectilinear grid of temperature is also used to calculate the change in the electrical conductivity which is an explicit function of temperature. This data is also mapped back into the finite element mesh and is used to change the electromagnetic sourcing characteristics. Augmented matrices are used to insure that calculations made on the geometric borders of the Femlab model are interpolated correctly.

Given the axial symmetry of the problem, we used a 2D-axisymmetric mesh consisting of 13,641 nodes and 26,880 elements. The Femlab 'Fldaspk' ordinary differential equation solver was used to achieve convergence. This is a robust variant of the traditional ODE15s stiff differential equation solver used in solving finite element problems in Matlab. Ablations were simulated at source voltages of 0, 2.5, 5, 7.5, 10, 12.5, 15, 17.5, 20, 22.5, 25, 27.5, and 30 volts. For each of the source voltages, we varied the initial level of tissue perfusion at 0%, 20%, 40%, 60%, 80%, and 100% normal tissue perfusion (6.4 × 10^-3 ^cubic meters of blood/ cubic meter of tissue/ second) [9]. Ambient tissue temperature was assumed to be 37°C. The model simulates a 15 minute ablation and updates tissue parameters at 2 second timestep intervals. Once the 15 minute ablation has ended, the model continues to solve solutions for 15 minutes post-ablation. For each simulation, the electric field (E), the current density (J), the temperature (T), and the tissue damage (D) were calculated. All calculations were implemented on a Dual 3.02 GHz Xeon processor workstation with 4 GByte RAM. Each simulation takes approximately 3 hours to run.

### Experimental Validation

To validate the computational model, experimental measurements were made in 6 freshly excised porcine liver sections. A single needle ablation probe with a 2 cm uninsulated tip was inserted 3 cm into each liver tissue. Since commercial RF ablation generators operate using either constant temperature or constant power feedback algorithms, an experimental constant voltage RF generator (500 kHz) was used [5]. Tissue samples were allowed to equilibrate to room temperature (approximately 22°C) prior to the start of ablation. Two samples were ablated at 20 volts for 15 minutes. After allowing the tissue to cool for an additional 15 minutes, the probes were removed and the tissue was bisected to expose the lesion. The tissues were immediately placed in a 1% 2,3,5-triphenyltetrazolium chloride (red) solution for 20 minutes to stain for tissues containing active dehydrogenase, an indicator of cell viability [58,59]. This stains healthy tissue brick red, leaving the ablated region a pale grey color. The maximum width and depth of the macroscopically pale ablated regions were measured. This procedure was repeated for two samples at 25 volts and for the last two samples at 30 volts.

Computational model calculations were made at 20, 25, and 30 volts following the same experimental protocol. Ambient temperature for these calculations was 22°C instead of the 37°C temperature used in the main simulations. The calculated lesion sizes were directly compared with the measurements in tissue.

## Results

Table 1 shows the maximum temperatures attained in tissue for the computational models for a range of voltages (2.5–30 Volts) and tissue perfusion rates (0–100% normal tissue perfusion.). The table shows a nonlinear relationship between the source voltage and the maximum temperature that results from the use of a temperature-dependent electrical conductivity. The maximum variation in the temperature data for a given source voltage did not exceed 17%. The data show that the rate of temperature increase accelerates as a function of the source voltage. As the level of tissue perfusion increases, tissue temperature decreases.

**Table 1:** Maximum Temperature (Degrees Celsius)^1 ^Values represent the maximum temperature attained in tissue for the computational models.

Source Voltage (Volts)	0%^2 ^Perfusion (0.0 × 10^-3 ^m_b_^3^/m_t_^3^/s)	20%^2 ^Perfusion (1.3 × 10^-3 ^m_b_^3^/m_t_^3^/s)	40%^2 ^Perfusion (2.6 × 10^-3 ^m_b_^3^/m_t_^3^/s)	60%^2 ^Perfusion (3.8 × 10^-3 ^m_b_^3^/m_t_^3^/s)	80%^2 ^Perfusion (5.1 × 10^-3 ^m_b_^3^/m_t_^3^/s)	100%^2 ^Perfusion (6.4 × 10^-3 ^m_b_^3^/m_t_^3^/s)
2.5	37.3	37.3	37.2	37.2	37.2	37.2
5.0	38.6	38.5	38.4	38.3	38.2	38.2
7.5	41.0	40.6	40.3	40.1	40.0	39.9
10.0	44.3	43.6	43.1	42.8	42.5	42.3
12.5	48.8	47.7	46.9	46.3	45.8	45.4
15.0	54.6	52.9	51.6	50.7	50.0	49.4
17.5	62.0	59.5	57.7	56.2	55.2	54.3
20.0	71.1	67.9	65.5	63.4	61.9	60.7
22.5	82.4	78.6	75.4	73.0	70.7	69.0
25.0	96.1	91.8	88.0	84.8	82.1	79.7
27.5	112.7^3^	107.8^3^	103.4^3^	99.5	96.1	93.4
30.0	132.5^3^	126.9^3^	121.8^3^	117.4^3^	113.4^3^	109.9^3^

^1 ^– The maximum temperature for the case of 0% perfusion was located along the center of the conducting electrode. In all other cases, the maximum temperature occurred at the tip of the probe; ^2 ^– The units for tissue perfusion are cubic meters of blood (m_b_^3^) per cubic meter of tissue (m_t_^3^) per second; ^3 ^– These temperatures do not account for energy loses associated with tissue desiccation or gas formation.

Table 2 shows the maximum electrical conductivity in the tissue after heating for 15 minutes at a variety of source voltages. All tissues initially have an electrical conductivity of 0.144 S/m at 37°C. The data show that tissue electrical conductivity is primarily a function of the source voltage, changing 320% over the course of a 15 minute ablation using a 30 volt source. With normal tissue perfusion (6.4 × 10^-3 ^m_b_^3^/ m_t_^3^/s), the electrical conductivity changes as much as 260% using a 30 volt source. The electrical conductivity is indirectly a function of tissue perfusion since tissue perfusion is zero in the necrosed treatment volume. Tissue perfusion lowers the tissue temperature outside the treatment volume which helps to conduct heat away from temperatures within the ablated area..

**Table 2:** Maximum Electrical Conductivity (Siemens/meter)^1 ^Values represent the maximum electrical conductivity attained in tissue for the computational models.

Source Voltage (Volts)	0%^2 ^Perfusion (0.0 × 10^-3 ^m_b_^3^/m_t_^3^/s)	20%^2 ^Perfusion (1.3 × 10^-3 ^m_b_^3^/m_t_^3^/s)	40%^2 ^Perfusion (2.6 × 10^-3 ^m_b_^3^/m_t_^3^/s)	60%^2 ^Perfusion (3.8 × 10^-3 ^m_b_^3^/m_t_^3^/s)	80%^2 ^Perfusion (5.1 × 10^-3 ^m_b_^3^/m_t_^3^/s)	100%^2 ^Perfusion (6.4 × 10^-3 ^m_b_^3^/m_t_^3^/s)
2.5	0.144	0.144	0.144	0.144	0.143	0.143
5.0	0.147	0.147	0.146	0.146	0.146	0.146
7.5	0.153	0.152	0.151	0.151	0.150	0.150
10.0	0.162	0.160	0.159	0.158	0.157	0.156
12.5	0.173	0.170	0.168	0.167	0.165	0.164
15.0	0.189	0.185	0.181	0.179	0.177	0.175
17.5	0.210	0.203	0.198	0.194	0.191	0.189
20.0	0.238	0.228	0.221	0.215	0.210	0.207
22.5	0.274	0.262	0.251	0.244	0.237	0.232
25.0	0.321	0.306	0.293	0.282	0.273	0.265
27.5	0.383	0.364	0.348	0.334	0.321	0.312
30.0	0.463	0.440	0.419	0.401	0.385	0.372

^1 ^– The maximum electrical conductivity for the case of 0% perfusion was located along the center of the conducting electrode. In all other cases, the maximum temperature occurred at the tip of the probe; ^2 ^– The units for tissue perfusion are cubic meters of blood (m_b_^3^) per cubic meter of tissue (m_t_^3^) per second.

Table 3 shows the maximum SAR computed for a range of voltages and tissue perfusion rates. The SAR is defined as SAR = σ /ρ*|E|^2^, where σ is the electrical conductivity, ρ is the tissue density, and |E| is the magnitude of the electric field. The data shows that the SAR is highest with increasing source voltage with no tissue perfusion. Initially, this seems counterintuitive as one would expect a higher maximum SAR for perfused flows, where a greater amount of power is needed to compensate for the convective heat loss. This observation can be explained by the large changes in the electrical conductivity (Table 2). Since higher temperatures are achieved for cases with no tissue perfusion, the change in the electrical conductivity is highest with no tissue perfusion. Since, at a given point, the density and the magnitude of the electric field are essentially constant (<0.02% change), the SAR will vary as a function of the electrical conductivity only.

**Table 3:** Maximum Specific Absorption Rate (Watts/kg)^1 ^Values represent the maximum specific absorption rate (SAR) attained in tissue for the computational models.

Source Voltage (Volts)	0%^2 ^Perfusion (0.0 × 10^-3 ^m_b_^3^/m_t_^3^/s)	20%^2 ^Perfusion (1.3 × 10^-3 ^m_b_^3^/m_t_^3^/s)	40%^2 ^Perfusion (2.6 × 10^-3 ^m_b_^3^/m_t_^3^/s)	60%^2 ^Perfusion (3.8 × 10^-3 ^m_b_^3^/m_t_^3^/s)	80%^2 ^Perfusion (5.1 × 10^-3 ^m_b_^3^/m_t_^3^/s)	100%^2 ^Perfusion (6.4 × 10^-3 ^m_b_^3^/m_t_^3^/s)
2.5	645.6	645.3	645.1	644.9	644.8	644.8
5.0	2608	2603	2600	2598	2596	2595
7.5	5966	5940	5924	5912	5903	5896
10.0	10850	10770	10720	10680	10650	10630
12.5	17450	17260	17120	17030	16950	16900
15.0	26030	25620	25330	25120	24970	24860
17.5	36940	36140	35600	35190	34900	34680
20.0	50590	49180	48230	47480	47000	46620
22.5	67470	65210	63520	62510	61610	60990
25.0	88170	84890	82360	80440	78940	77910
27.5	113300	108900	105300	102400	100100	98390
30.0	143400	137700	133000	129000	125700	123000

^1 ^– The maximum current density was always located at the tip of the ablation probe. Therefore, all values listed above are comparable with each other; ^2 ^– The units for tissue perfusion are cubic meters of blood (m_b_^3^) per cubic meter of tissue (m_t_^3^) per second.

**Figure 4:**
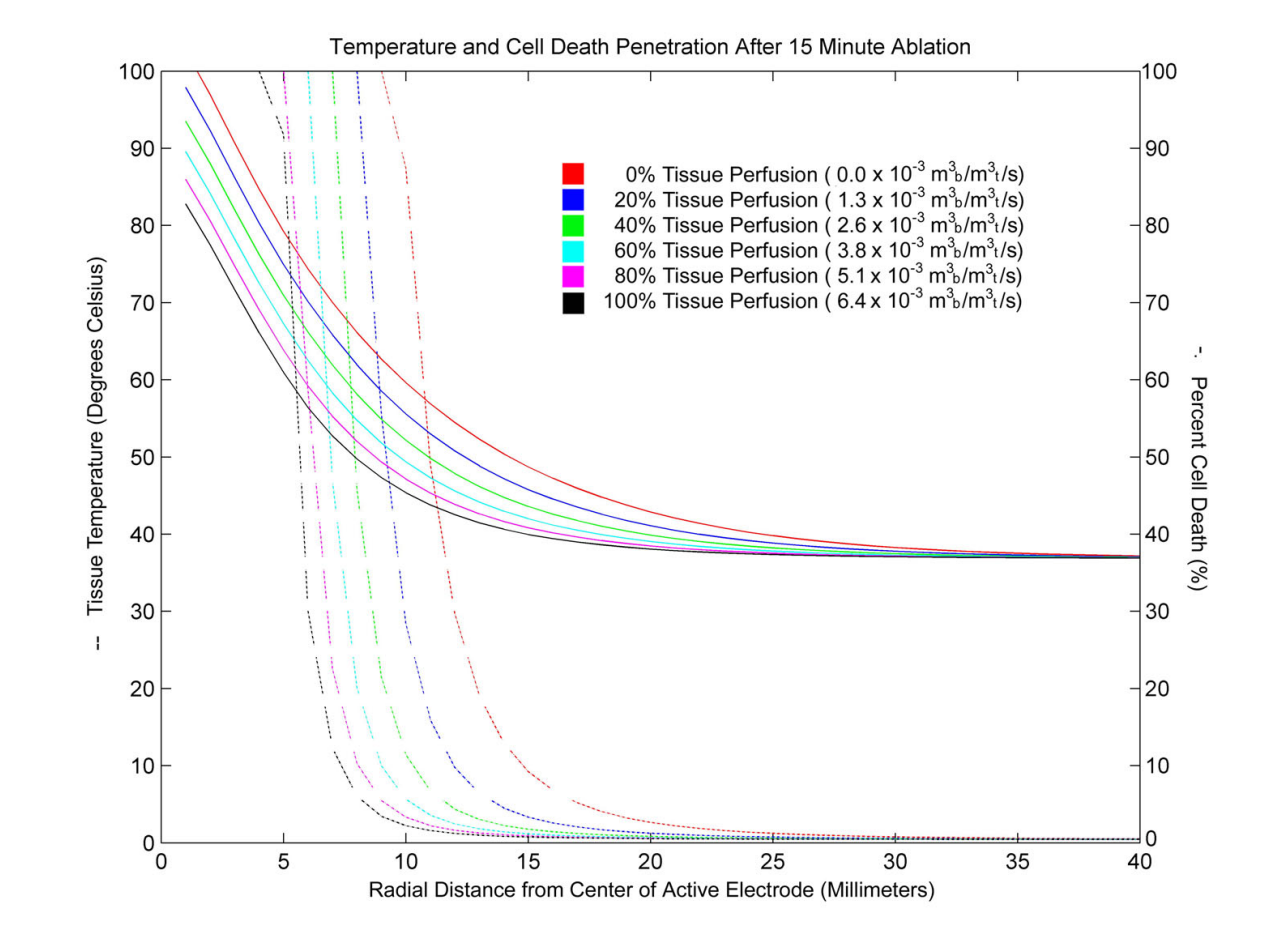
Tissue temperature and cell death penetration for a 15 minute ablation using a 30 volt constant voltage source. Simulation results for a 15 minute ablation using a 30 volt constant voltage source measured from the center of the active electrode. The graph shows temperature (solid) and cell death (dotted) penetration into liver tissue for a range of tissue perfusion rates. The units for tissue perfusion are cubic meters of blood (m_b_^3^) per cubic meter of tissue (m_t_^3^) per second (s).

Figure 4 shows the tissue temperature and the cell death penetration into tissue for a 15 minute ablation using a 30 volt constant voltage source for perfusion rates ranging from no perfusion to 100% normal tissue perfusion. The data show that cell death decreases more rapidly than tissue temperature. At the center of the active electrode, temperatures decrease as a function of the inverse of the radius squared (1/r^2^), whereas cell damage exhibits an S-shaped curve. Figure 4 shows that 63% tissue damage is roughly correlated with the 60°C isotherm for liver tissues. Conventional temperature isotherms for tissue damage for hyperthermia (42°C) and radiofrequency ablation (47°C) substantially overestimate the size of the lesions.

**Figure 5:**
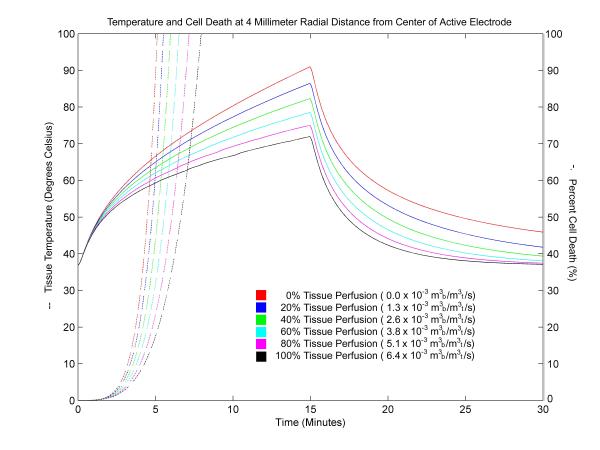
Tissue temperature and cell death at a distance of 4 millimeters from the center of the active electrode using a 30 volt constant voltage source. Ablation simulation results attained 4 millimeters from the center of the active electrode for a 15 minute ablation using a 30 volt constant voltage source. The graph shows temperature (solid) and cell death (dotted) penetration into liver tissue for a range of tissue perfusion rates. The units for tissue perfusion are cubic meters of blood (m_b_^3^) per cubic meter of tissue (m_t_^3^) per second (s).

**Figure 6:**
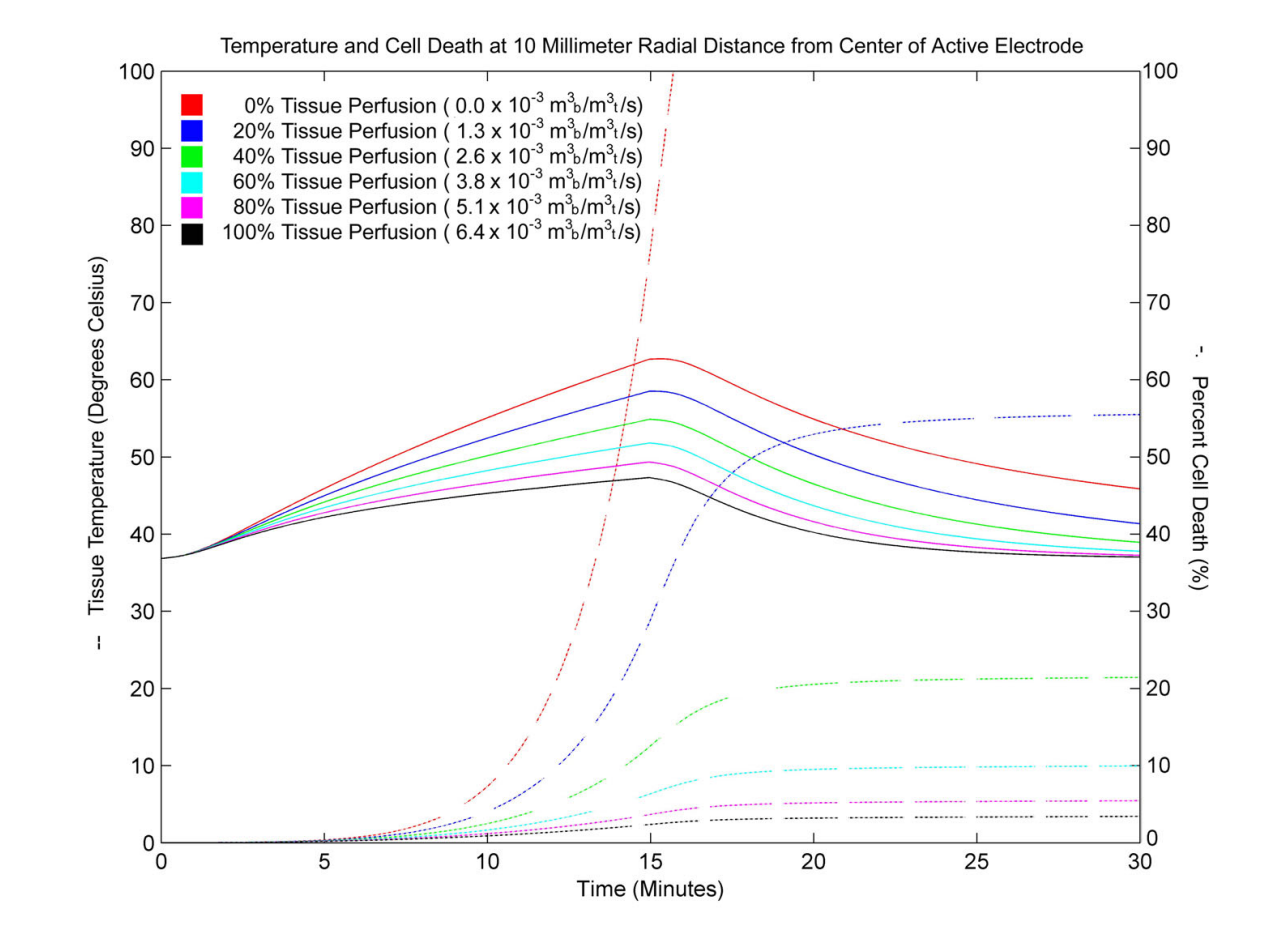
Tissue temperature and cell death at a distance of 10 millimeters from the center of the active electrode using a 30 volt constant voltage source. Ablation simulation results attained 10 millimeters from the center of the active electrode for a 15 minute ablation using a 30 volt constant voltage source. The graph shows temperature (solid) and cell death (dotted) penetration into liver tissue for a range of tissue perfusion rates. The units for tissue perfusion are cubic meters of blood (m_b_^3^) per cubic meter of tissue (m_t_^3^) per second (s).

Figure 5 shows a plot of tissue temperature and cell damage calculated at a distance of 4 millimeters from the center of the active electrode for a 15 minute ablation using a 30 volt constant voltage source. Temperature decrease and cell damage that occurs after the ablation is monitored for an additional 15 minutes. The data show that near the electrode, tissue damage will reach 100% well within the first few minutes of energy application. For cases of no tissue perfusion, 100% tissue damage occurs after 5 minutes at a distance of 4 millimeters. For cases with normal tissue perfusion, 100% tissue damage occurs approximately 8 minutes into the ablation. At a distance of 10 millimeters from the center of the active electrode under the same conditions (Figure 6), the data show that tissue damage will not always reach 100%. For the case of no perfusion, 100% cell damage is reached a minute after the termination of radiofrequency energy. In cases with varying levels of tissue perfusion, cell damage is significantly reduced and, in some cases, insignificant. Although the overall temperatures are lower at 10 millimeters than at 4 millimeters, temperatures near 60°C are reached but do not result in complete tissue damage because the length of time in which the tissue is exposed is not sufficient.

**Figure 7:**
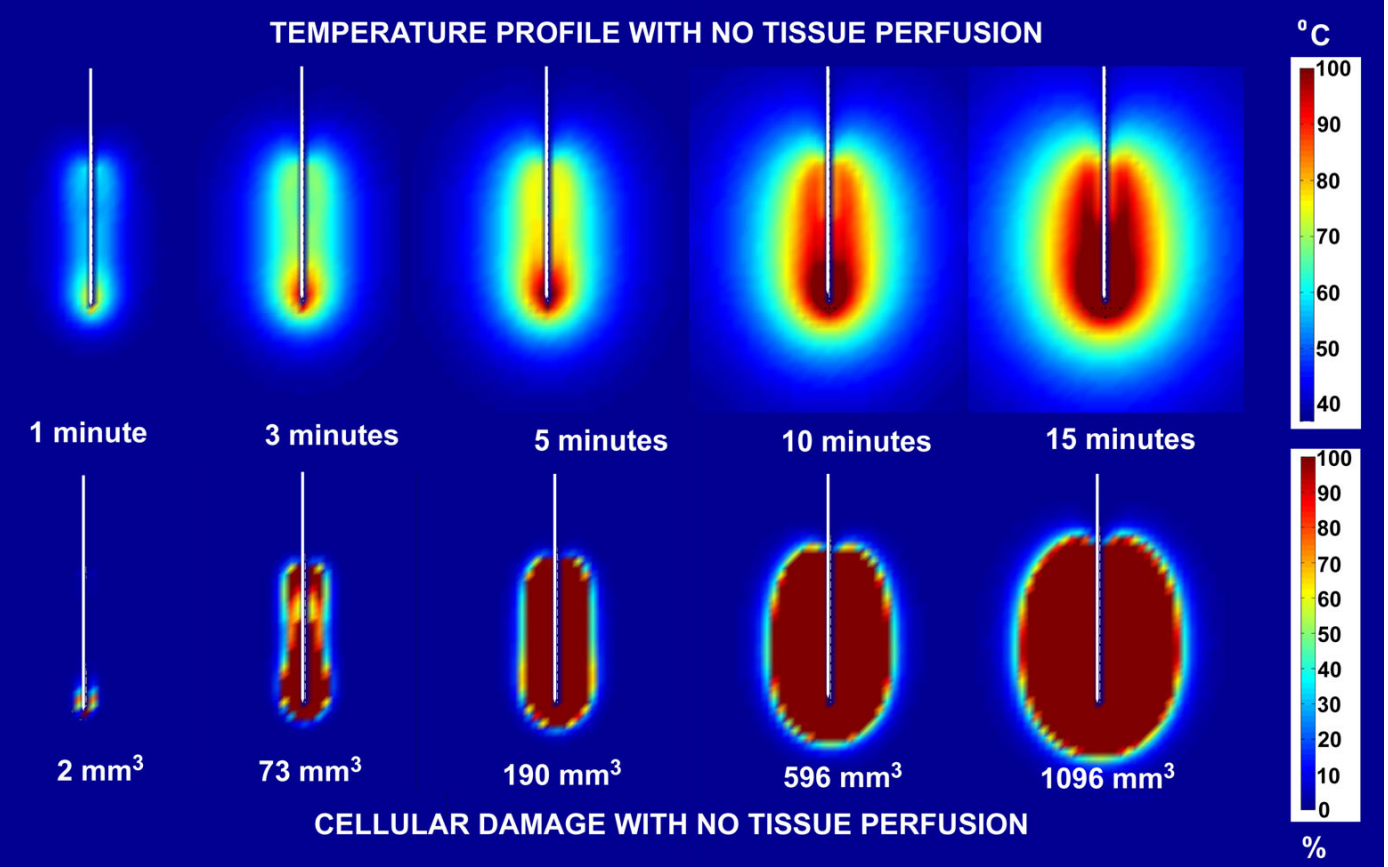
Comparison of temperature and lesion size development with no tissue perfusion for a 30 volt constant voltage source ablation. Ablation simulation results for a 30 volt constant voltage source ablation with no tissue perfusion. The results on the top half of the figure represent the temperature distribution surrounding the ablation probe in degrees Celsius. The results on the bottom half of the figure represent the percent tissue damage. The numbers listed at the bottom are the lesion volume sizes computed from the 63% cell damage isocontours at each time interval shown.

**Figure 8:**
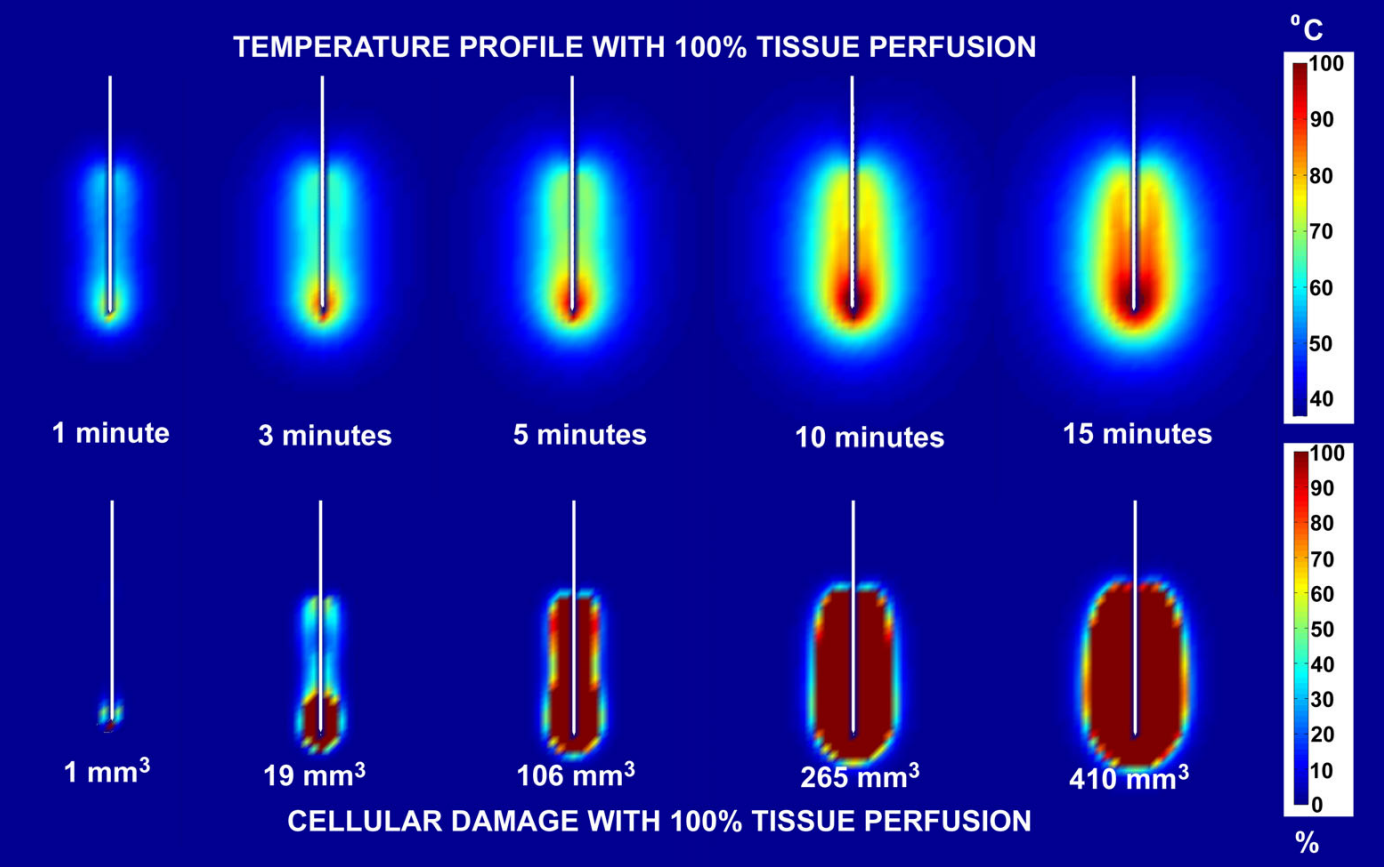
Comparison of temperature and lesion size development with normal tissue perfusion (6.4 × 10^-3 ^m_b_^3^/m_t_^3^/s) for a 30 volt constant voltage source ablation. Ablation simulation results for a 30 volt constant voltage source ablation with normal tissue perfusion (6.4 × 10^-3 ^m_b_^3^/m_t_^3^/s). The results on the top half of the figure represent the temperature distribution surrounding the ablation probe in degrees Celsius. The results on the bottom half of the figure represent the percent tissue damage. The numbers listed at the bottom are the lesion volume sizes computed from the 63% cell damage isocontours at each time interval shown.

**Figure 9:**
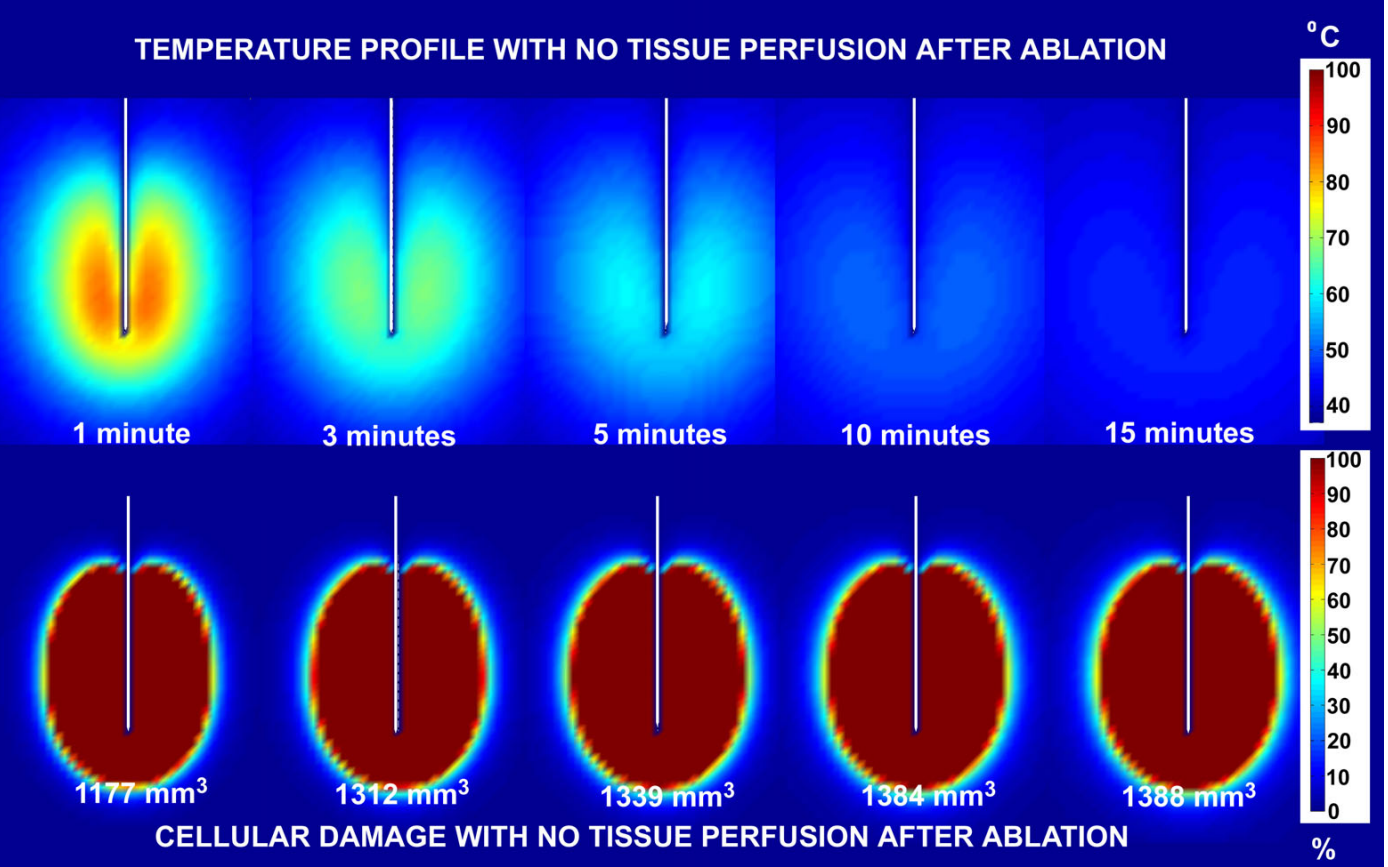
Comparison of temperature and lesion size development post ablation with no tissue perfusion for a 30 volt constant voltage source ablation. Ablation simulation results following a 15 minute ablation without perfusion for a 30 volt constant voltage source ablation. The results on the top half of the figure represent the temperature distribution surrounding the ablation probe in degrees Celsius. The results on the bottom half of the figure represent the percent tissue damage. The numbers listed at the bottom are the lesion volume sizes computed from the 63% cell damage isocontours at each time interval shown.

**Figure 10:**
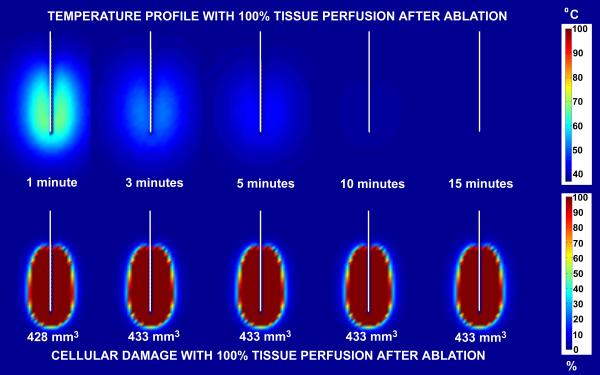
Comparison of temperature and lesion size development post ablation with normal tissue perfusion (6.4 × 10^-3 ^m_b_^3^/m_t_^3^/s) for a 30 volt constant voltage source ablation. Ablation simulation results following a 15 minute ablation with normal tissue perfusion (6.4 × 10^-3 ^m_b_^3^/m_t_^3^/s) for a 30 volt constant voltage source ablation. The results on the top half of the figure represent the temperature distribution surrounding the ablation probe in degrees Celsius. The results on the bottom half of the figure represent the percent tissue damage. The numbers listed at the bottom are the lesion volume sizes computed from the 63% cell damage isocontours at each time interval shown.

Figure 7 and 8 show comparisons of temperature distribution and lesion size development with no tissue perfusion (Figure 7) and with normal tissue perfusion (Figure 8) for a 30 volt constant voltage source ablation at 1, 3, 5, 10 and 15 minutes. The data demonstrate that the shapes of the temperature isotherms do not correlate well with tissue damage profiles. Tissue perfusion greatly affects the size of the resulting ablated region. At 15 minutes, lesion volumes are 267% larger without perfusion than with tissue perfusion. Figures 9 and 10 show a comparison of the temperature distribution and lesion size development with no tissue perfusion (Figure 9) and with normal tissue perfusion (Figure 10) at 1, 3, 5, 10, and 15 minutes following a 15 minute constant 30 volt ablation. In the case of no tissue perfusion, the lesion size continues to grow 14% within the first 5 minutes after radiofrequency energy is terminated. The lack of tissue perfusion prolongs the time needed to conduct the heat away from tissues near the surface of the ablation electrode. For cases with normal tissue perfusion, heat is quickly dissipated by tissue perfusion causing the lesion volume to stabilize in less than 2 minutes. By definition, the area of coagulative necrosis has no tissue perfusion. This accounts for the residual heating pattern within the ablated region as seen up to 3 minutes following the ablation.

A comparison of lesion volumes with no tissue perfusion computed using 63% and 100% iso-damage threshold contours and 42°C, 47°C, 60°C, and 90°C isothermal contours is presented for the cases of no tissue perfusion (Table 4) and normal tissue perfusion (Table 5). The sensitivity of the cell damage function (Figure 4) results in less than 10% differences in the size of lesions calculated using tissue damage thresholds of 63% and 100% cell damage. In contrast, volume sizes based on isothermal contours varies considerably at each temperature. When using traditional isothermal contours of 42°C and 47°C, the calculated lesion volumes are grossly overestimated by 500% and 167%, respectively. In both the case of no tissue perfusion and normal tissue perfusion, the 60°C isothermal contour resembles the lesion sizes calculated using the iso-damage contours.

**Table 4:** Lesion Volume with No Tissue Perfusion Values represent the total volume of tissue necroses calculated over the course of the simulated ablation using various cell damage thresholds (D) and isothermal temperatures (IT).

Source Voltage (Volts)	D = 63% (mm^3^)	D = 100% (mm^3^)	IT = 42°C (mm^3^)^1^	IT = 47°C (mm^3^)^1^	IT = 60°C (mm^3^)	IT = 90°C (mm^3^)
2.5	0	0	0	0	0	0
5.0	0	0	0	0	0	0
7.5	0	0	0	0	0	0
10.0	0	0	89	0	0	0
12.5	0	0	444	13	0	0
15.0	0	0	942	216	0	0
17.5	9	3	1649	526	6	0
20.0	121	67	2508	915	87	0
22.5	314	242	3549	1414	296	0
25.0	577	495	4860	2070	547	6
27.5	923	809	6452	2866	870	55
30.0	1388	1228	8386	3830	1243	202

^1 ^– The 42°C and 47°C isothermal volumes were chosen specifically because they are frequently used to establish damage thresholds in hyperthermia and radiofrequency ablation, respectively.

**Table 5:** Lesion Volume with 100% Normal Tissue Perfusion. Values represent the total volume of tissue necroses calculated over the course of the simulated ablation using various cell damage thresholds (D) and isothermal temperatures (IT).

Source Voltage (Volts)	D = 63% (mm^3^)	D = 100% (mm^3^)	IT = 42°C (mm^3^)^1^	IT = 47°C (mm^3^)^1^	IT = 60°C (mm^3^)	IT = 90°C (mm^3^)
2.5	0	0	0	0	0	0
5.0	0	0	0	0	0	0
7.5	0	0	0	0	0	0
10.0	0	0	1	0	0	0
12.5	0	0	47	0	0	0
15.0	0	0	185	6	0	0
17.5	0	0	358	68	0	0
20.0	4	2	513	177	1	0
22.5	23	19	784	287	15	0
25.0	131	89	1068	488	107	0
27.5	251	222	1502	759	241	3
30.0	433	364	2014	1064	423	24

^1 ^– The 42°C and 47°C isothermal volumes were chosen specifically because they are frequently used to establish damage thresholds in hyperthermia and radiofrequency ablation, respectively.

Table 6 shows a comparison of lesion width and depth computed using 63% and 100% iso-damage threshold contours and 42°C, 47°C, 60°C, and 90°C isothermal contours. The data show overestimations of 30–77% in the width and 18–54% in the depth of lesions when using traditional isothermal temperatures for a 15 minute ablation with no perfusion. Table 7 shows that in cases with normal tissue perfusion, calculations using traditional isothermal contours results in overestimations of 25–88% in the width and 15–41% in the depth of lesions.

**Table 6:** Lesion Dimensions with no Tissue Perfusion Values represent the maximum lesion width and depth calculated over the course of the simulated ablation using various cell damage thresholds (D) and isothermal temperatures (IT).

	Width (mm)						Depth (mm)					
Source Voltage (Volts)	D = 63%	D = 100%	IT = 42°C^1^	IT = 47°C^1^	IT = 60°C	IT = 90°C	D = 63%	D = 100%	IT = 42°C^1^	IT = 47°C^1^	IT = 60°C	IT = 90°C

2.5	0	0	0	0	0	0	0	0	0	0	0	0
5.0	0	0	0	0	0	0	0	0	0	0	0	0
7.5	0	0	0	0	0	0	0	0	0	0	0	0
10.0	0	0	8	0	0	0	0	0	19	0	0	0
12.5	0	0	16	0	0	0	0	0	26	0	0	0
15.0	0	0	22	8	0	0	0	0	31	13	0	0
17.5	6	4	28	14	4	0	7	5	35	24	6	0
20.0	10	8	32	18	10	0	21	16	39	28	18	0
22.5	14	12	36	22	14	0	25	24	43	31	24	0
25.0	18	18	40	26	18	4	28	26	45	34	28	6
27.5	22	22	44	30	22	8	31	30	49	37	30	14
30.0	26	26	46	34	26	12	33	33	51	39	33	23

^1 ^– The 42°C and 47°C isothermal volumes were chosen specifically because they are frequently used to establish damage thresholds in hyperthermia and radiofrequency ablation, respectively.

**Table 7:** Lesion Volume with 100% Normal Tissue Perfusion Values represent the maximum lesion width and depth calculated over the course of the simulated ablation using various cell damage thresholds (D) and isothermal temperatures (IT).

	Width						Depth					
Source Voltage (Volts)	D = 63%	D = 100%	IT = 42°C^1^	IT = 47°C^1^	IT = 60°C	IT = 90°C	D = 63%	D = 100%	IT = 42°C^1^	IT = 47°C^1^	IT = 60°C	IT = 90°C

2.5	0	0	0	0	0	0	0	0	0	0	0	0
5.0	0	0	0	0	0	0	0	0	0	0	0	0
7.5	0	0	0	0	0	0	0	0	0	0	0	0
10.0	0	0	2	0	0	0	0	0	2	0	0	0
12.5	0	0	8	0	0	0	0	0	16	0	0	0
15.0	0	0	10	0	0	0	0	0	25	0	0	0
17.5	0	0	14	6	0	0	0	0	27	7	0	0
20.0	4	4	16	8	2	0	5	4	29	23	2	0
22.5	6	6	20	10	6	0	10	8	31	25	8	0
25.0	10	8	24	14	8	0	23	23	33	27	23	0
27.5	12	12	26	18	12	4	25	25	36	29	25	5
30.0	16	14	30	20	16	6	27	27	38	31	27	9

^1 ^– The 42°C and 47°C isothermal volumes were chosen specifically because they are frequently used to establish damage thresholds in hyperthermia and radiofrequency ablation, respectively.

**Figure 11:**
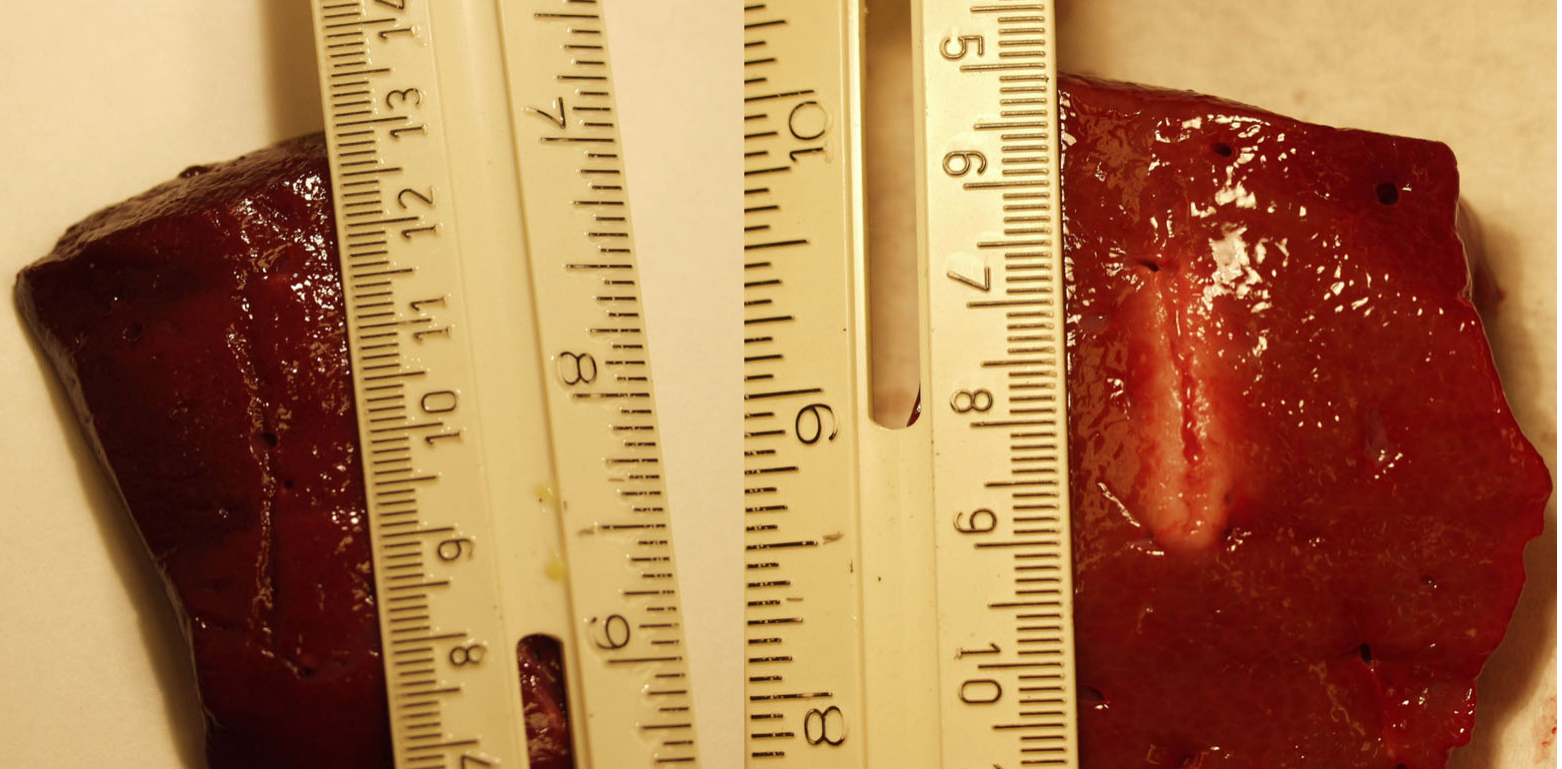
Experimental validation radiofrequency ablation lesions in excised porcine liver tissue produced by a 20 volt (left) and 30 volt (right) constant voltage radiofrequency generator (500 kHz) for a 15 minute exposure time. The lesions were produced using a single needle ablation probe with a 2 centimeter uninsulated tip.

To validate the computational model, ablation experiments were performed at room temperature (22°C) in excised porcine liver tissue using 20, 25, and 30 volt constant voltage radiofrequency sources (500 kHz). Ablations were made for a 15 minute exposure time. Figure 11 shows that no visible lesion can be seen in tissues where the 20 volt constant voltage ablation was performed, as predicted by the computational simulation. A lesion that was approximately 10 millimeters in width and 22 mm in depth resulted from the 30 volt constant voltage ablation. Table 8 shows a high correlation between the computational data calculated at 22°C and the experimental results.

**Table 8:** Comparison of Computational Data to Experimental Validation Data at 22°C.

	Lesion Width		Lesion Depth	
Source Voltage (Volts)	D = 63% (mm)	Experimental (mm)	D = 63% (mm)	Experimental (mm)

20.0	0	0	0	0
22.5	0	--	0	--
25.0	4	5	5	6
27.5	8	--	9	--
30.0	10	10	21	22

Simulated lesion dimensions with no tissue perfusion at an ambient temperature of 22°C using a 63% cell damage threshold (D) were compared to experimental measurements made at 20, 25, and 30 volts using an experimental constant voltage radiofrequency ablation source. Lesions were generated with a single needle ablation probe with a 2.0 cm active electrode. All lesion measurements were measured visually under a micrsope at 10× magnification.

## Discussion

To date, several computational studies have been performed to described the rate of lesion growth in radiofrequency ablation applications. In many cases, these studies use surrogate endpoints such as temperature isotherms and thermal dosing to calculate equivalent expressions for lesion size. While many models exists that account for far-more elaborate parameters such as tissue perfusion through large blood vessels, the interpretation of such models is difficult since most do not account for transient changes in tissue properties and often report tissue temperature only [6-9,12,14-19,56,57,60]. Several studies have identified that both exposure time and temperature contribute to tissue damage, however, few have actually calculated tissue damage. Those that do, have not allowed tissue damage to transiently influence the electrical and thermal properties of tissues [6,56].

In this study, we created a computational simulation that tested some of the basic assumptions made in modeling lesion growth problems. We developed a model where tissue perfusion and the electrical conductivity are allowed to vary at each time step and spatial position as a function of tissue damage and temperature. These simulations are significantly more time-consuming since gross simplifications to heating mechanisms are not made. Although our model geometry is simpler than others that appear in the literature, we chose to ignore large vessels since their position and impact are highly variable. We chose a simpler geometry so that the impact of damage-dependent tissue perfusion and temperature-dependent electrical conductivity could be assessed more directly.

The damage-dependent tissue perfusion accounts for physiological observations of tissue coagulation and local cessation of blood flow. Unlike thermal dosing, where thermal injury is calculated globally over the entire duration of an ablation, tissue damage is calculated at every time step. The intermediate tissue damage that results at every timestep influences the local tissue perfusion and creates a moving boundary condition which changes the local heat sink properties. Ignoring the intermediate timesteps causes tissue perfusion to remain constant throughout the entire ablation, which results in an underestimation of the true lesion size. The use of temperature-dependent electrical conductivity greatly affects modeling results, as the electrical conductivity has been shown to increase dramatically over the course of tissue heating [57]. When constant electrical conductivity is used, the SAR is grossly underestimate, which also results in an underestimation in lesion size.

An important outcome of this study is the demonstration that, temperature isotherms and tissue damage patterns are not synonymous. Traditional use of temperature isotherms that are used to define lesion size rely on coagulation temperature for protein (42–47°C) and grossly overestimate lesion dimensions. Our studies show that temperature decrease is gradual, while tissue damage decreases rapidly as a function of distance. It is this sharp decrease in tissue damage that causes lesion boundaries to appear fuzzy, as predicted by our model. The results also demonstrate that ablation lesions continue to grow after the applied power is terminated. Lesions continue to grow while temperature envelopes collapse after ablation since sufficiently high temperature are present to accrue tissue damage. In nearly all cases, lesions continued to grow several minutes following the ablation. A comparison of the resulting lesion dimensions between fully perfused and non-perfused tissues show that the lesion width decreases 38–46% and the lesion depth decreases 18–20% when tissue perfusion is accounted for in the model. Previous studies have shown that tissue perfusion can account for as much as 50% change in the size of the lesions generated during ablation [59].

An important observation in this study is the resemblance of the 60°C isocontour to lesion size. While the 42°C and the 47°C isotemperature contours are poor indicators of lesion size, 60°C is highly correlated with the lesion volumes. Seemingly, this would suggest that time-intensive tissue damage calculations need not be made since a critical temperature of 60°C can be used to identify lesion size. However, this is only true if the calculated temperature is a function of both transient changes in tissue perfusion and the electrical conductivity. In the absence of either of these phenomena, lesion sizes calculated at 60°C would underestimate lesion size.

The validation data demonstrate that the model accurately accounts for the behavior of lesion growth in tissue. There are, however, a few limitations to this model. First, it is well established that temperature elevation of tissues results in the denaturing of proteins, which may drastically change the electrical conductivity of tissue in a nonlinear fashion [51,57]. Preliminary data suggests that the electrical conductivity substantially increases, which would likely increase the rate of tissue damage. The results of this study show that as a first order approximation the conductivity of equivalent sodium chloride solutions produces results that are within 5% of the experimental measurements. Although the phenomena described in this reporting are applicable to different tissues, the resulting lesion dimensions and temperature profiles in this study apply only to liver tissue. Similar studies can be made in other tissues, but were not pursued in this study.

A second limitation in our model is that it is only valid for temperatures below 100°C. At temperatures above 100°C, tissues begin to boil and generate gas. When this occurs, some of the energy that contributes to temperature increase is used to change the water content of tissues into gas. At substantially higher temperatures, the composition of gas may be highly complex as tissue begins to burn and break down. Although gas generation is commonly seen in clinical use of radiofrequency ablation, impedance rises due to tissue charring limit the progressive rise in temperature. The complexity of multi-phasic ablation was beyond the scope of this study.

## Disclaimer

The mention of commercial products, their sources, or their use in connection with material reported herein is not to be construed as either an actual or implied endorsement of such products by the Department of Health and Human Services.
